# Coronary artery spasm complicated by atrial septal puncture: A case report

**DOI:** 10.1097/MD.0000000000043679

**Published:** 2025-08-01

**Authors:** Ping Gong, Yong Da Zhang, Yuan Zhi Zhou, Tao Zhou

**Affiliations:** a Mianyang 404 Hospital, Mianyang, China.

**Keywords:** atrial fibrillation, atrial septal puncture, coronary artery spasm, radiofrequency ablation

## Abstract

**Rationale:**

The occurrence of ST-segment elevation on electrocardiogram (ECG) during transseptal puncture in atrial fibrillation procedures is relatively uncommon, and its pathogenesis remains unclear. In this report, we clearly identified severe coronary artery spasm during atrial septal puncture as one of the causes of ECG ST-segment elevation.

**Patient concerns:**

A 66-year-old woman was admitted to the hospital due to “paroxysmal palpitations for 1 year, with worsening symptoms for 1 week.” Both the admission ECG and previous ECGs diagnosed her with atrial fibrillation. Immediately after routine atrial septal puncture, she experienced a drop in blood pressure and abnormal ECG changes, leading to serious complications.

**Diagnoses:**

Persistent atrial fibrillation.

**Interventions:**

Femoral artery puncture for coronary angiography indicated occlusion of the proximal right coronary artery. Percutaneous coronary intervention of the right coronary artery was immediately performed. During the operation, it was confirmed that there was combined spasm on the basis of coronary artery stenosis. Nitroglycerin and sodium nitroprusside were repeatedly intravenously injected, norepinephrine was intravenously infused, an intra-aortic balloon pump and a temporary pacemaker were implanted. Subsequently, a 4.0 × 18 mm coronary stent was implanted.

**Outcomes:**

The patient’s symptoms improved, with resolution of ST-segment elevation on the ECG, and blood pressure and heart rate increased. Ultimately, the patient was discharged after 12 days of hospitalization with improvement.

**Lessons:**

Coronary artery spasm after atrial septal puncture is rare, and most cases are transient and do not lead to serious complications. However, if the patient has concurrent fixed stenosis, it may cause persistent vasospasm, resulting in severe complications. Preoperative coronary angiography or coronary CTA is a necessary examination.

## 1. Introduction

Atrial fibrillation is an age-related disease, and currently ablation surgery is a common treatment method.^[1]^ Regardless of whether it is radiofrequency ablation, cryoablation, or pulsed ablation, atrial septal puncture is a fundamental step in the procedure. During the operation, atrial septal puncture may lead to complications. This report details a severe complication caused by coronary artery spasm after atrial septal puncture.

## 2. Case report

**Figure 1.**
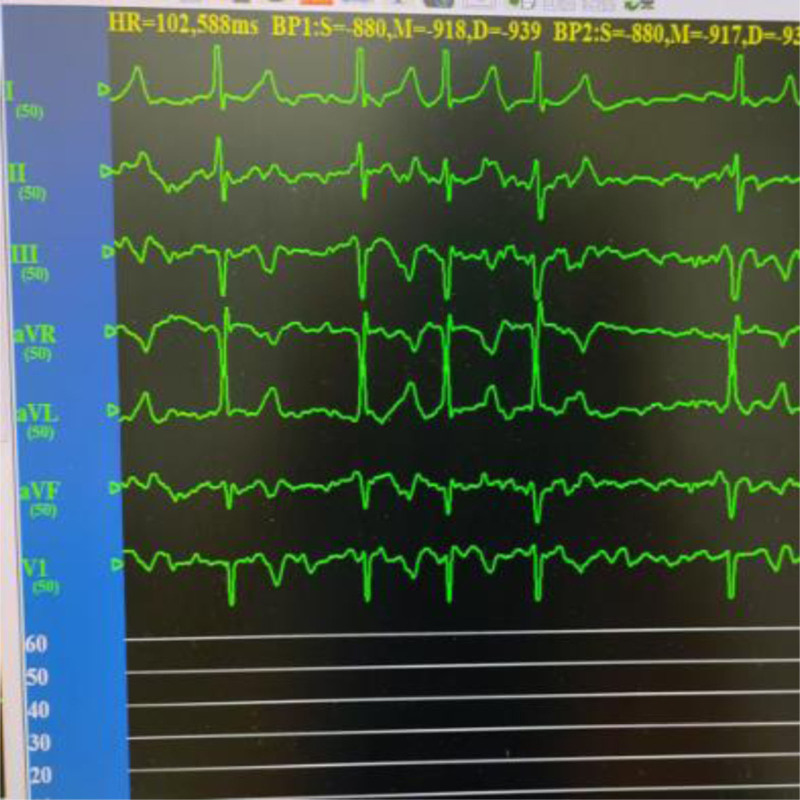
Electrocardiogram shows atrial fibrillation.

**Figure 2.**
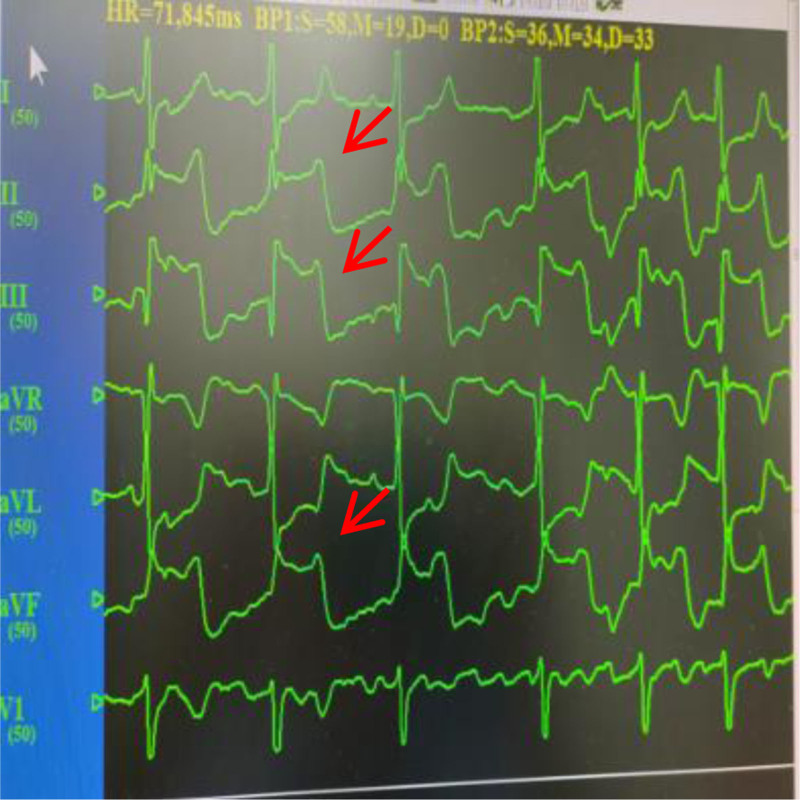
ST segment elevation in inferior wall leads of electrocardiogram.

**Figure 3.**
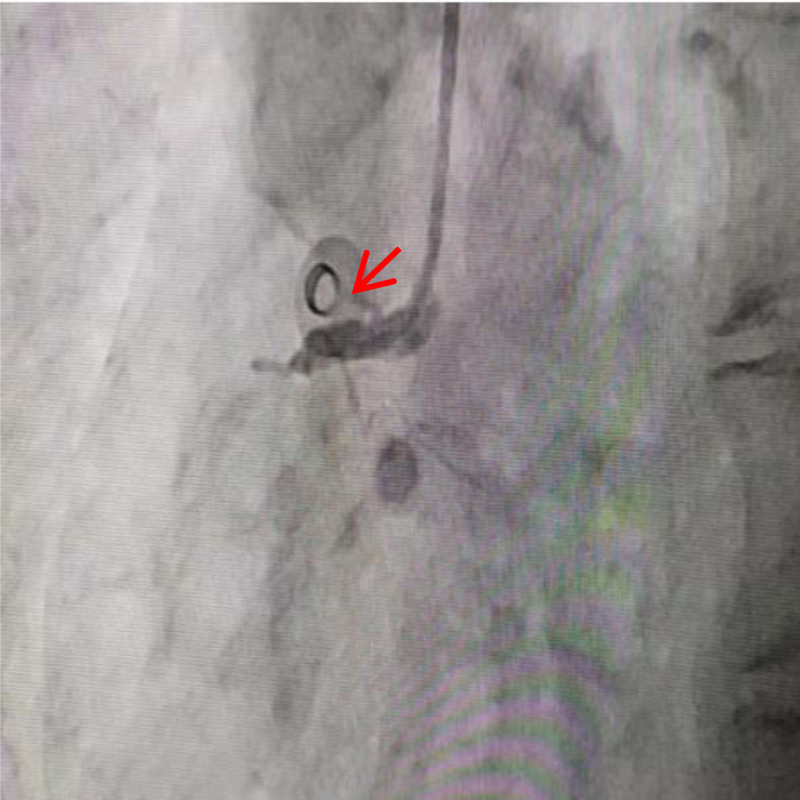
Coronary angiography with right coronary occlusion.

**Figure 4.**
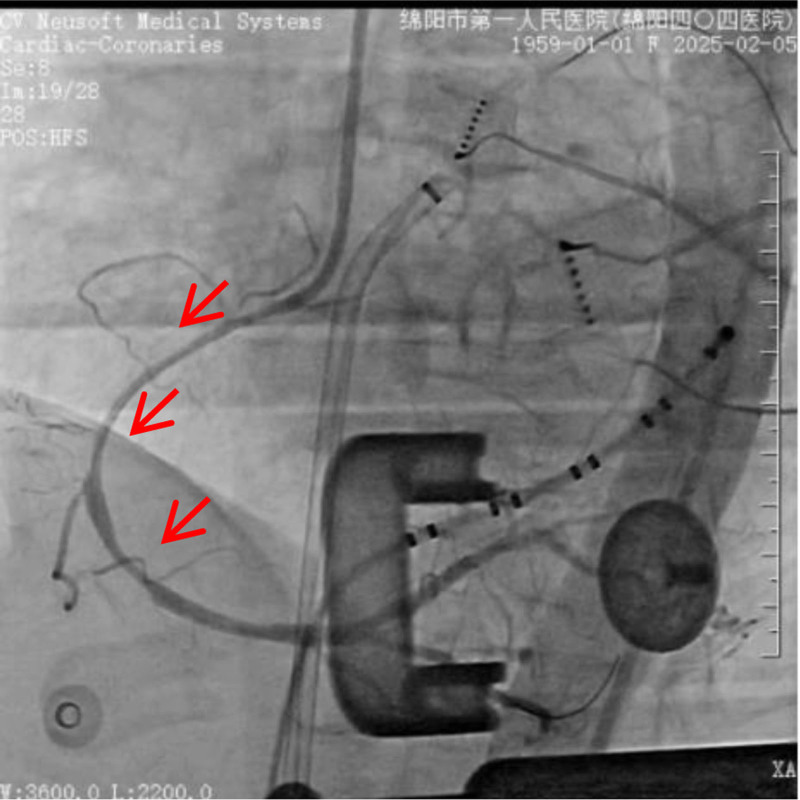
Coronary artery spasm.

**Figure 5.**
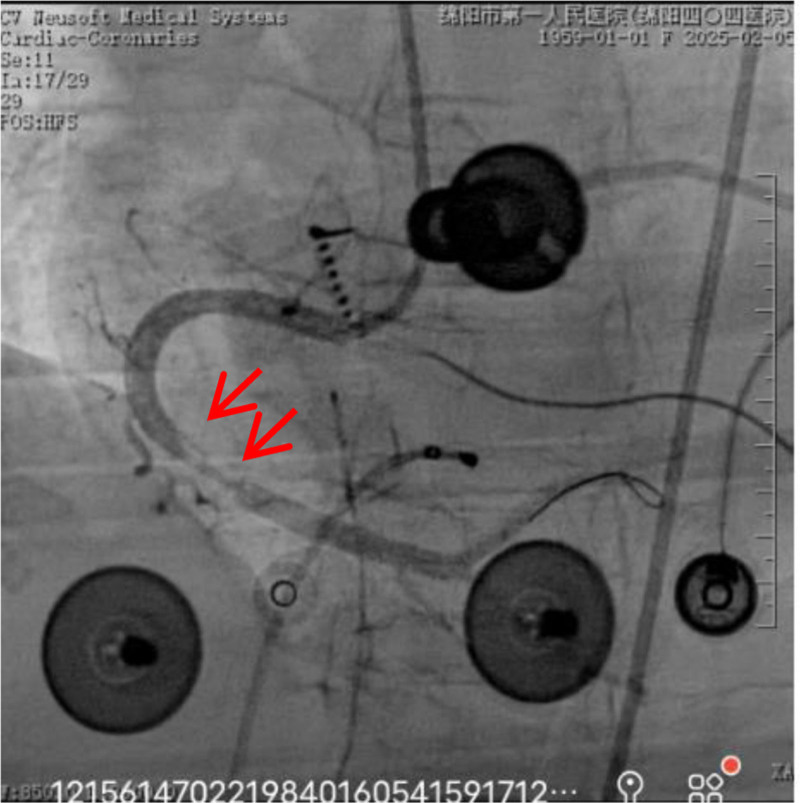
There is a fixed stenosis after the relief of right coronary spasm.

**Figure 6.**
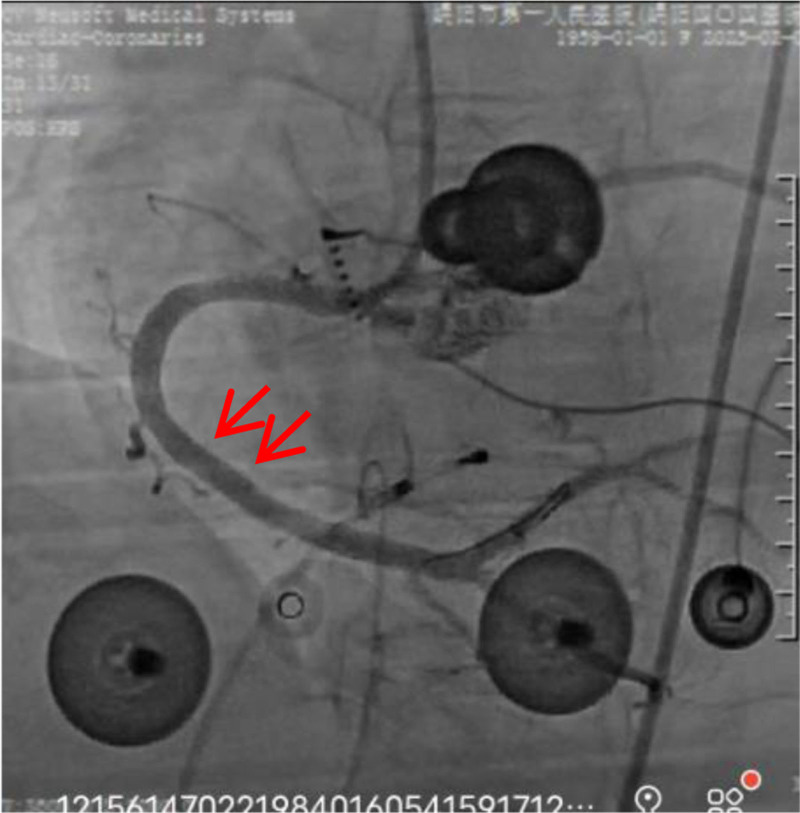
Narrowing relieved after implantation of a stent.

**Figure 7.**
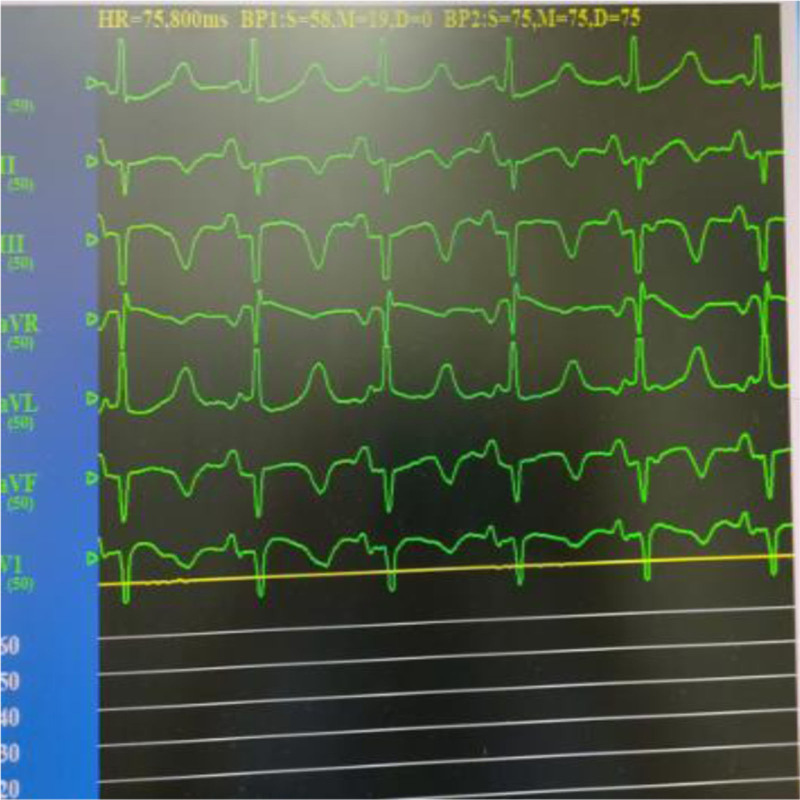
ECG ST segment returns to normal. ECG = electrocardiogram.

The patient is a 66-year-old female who was admitted on January 7, 2025, due to “paroxysmal palpitations for 1 year, with worsening symptoms for 1 week.” She was diagnosed with atrial fibrillation 1 year ago and was admitted for atrial fibrillation ablation surgery due to worsening palpitations 1 week prior. She denied having underlying diseases such as hypertension, diabetes, and coronary heart disease, and had no bad habits like smoking or heavy alcohol consumption. Physical examination upon admission: body temperature (T) 36.2 °C, pulse rate (P) 92 beats per minute, respiratory rate (R) 20 breaths per minute, blood pressure (BP) 113/73 mm Hg; heart rate 114 beats per minute, arrhythmia, the intensity of the first heart sound varied, and there was a pulse deficit. The electrocardiogram (ECG) showed atrial fibrillation (Fig. 1). Transthoracic echocardiography revealed a left ventricular ejection fraction of 0.68 and a left atrial anteroposterior diameter of 33 mm. Transesophageal echocardiography performed within 24 hours prior to the procedure excluded the presence of left atrial appendage thrombus. The patient was fasted for at least 8 hours preoperatively. Under the induction of anesthesia with propofol, sufentanil, and vecuronium bromide, general anesthesia was maintained with continuous infusion of propofol and remifentanil. In the right anterior oblique 45° position, transseptal puncture was performed using an 8F APT introducer sheath and puncture needle. After the sheath crossed the atrial septum, 6000 units of heparin (100 u/kg) were administered to prevent thrombosis. Following successful atrial septal puncture, a drop in BP was observed, and ST-segment elevation was noted in the inferior wall leads (II, III, aVF) on electrocardiographic monitoring (Fig. 2). Immediate femoral artery puncture for coronary angiography revealed occlusion of the proximal segment of the right coronary artery (Fig. 3). Percutaneous coronary intervention was performed on the right coronary artery, and coronary artery spasm was considered intraoperatively (Fig. 4). Nitroglycerin and sodium nitroprusside were repeatedly administered intravenously, but the effect of norepinephrine in raising BP was not satisfactory. Following the immediate implantation of an intra-aortic balloon pump and a temporary pacemaker, and multiple intravenous administrations of nitroglycerin, the patient’s vascular spasm resolved, with the vessel diameter expanding to 4.0 mm. However, an 85% stenosis was found in the mid-segment, prompting the insertion of a 4.0 × 18 mm coronary stent. The patient’s symptoms improved, the ST-segment elevation on the ECG decreased (Figs. 5, 6, 7), BP and heart rate increased, and then the patient was transferred to the intensive care unit of the hospital. In the intensive care unit, the patient experienced repeated ventricular fibrillation. Treatments such as deep sedation, anti-arrhythmic drugs, and mild hypothermia were given. Finally, the patient was discharged in improved condition after 12 days of hospitalization. This case obtained informed consent from the patient and their children.

## 3. Conclusion

During the entire surgical procedure for atrial fibrillation, situations that can lead to ST-segment elevation on the ECG include: 1. The use of anesthetic, sedative, and analgesic medications; 2. During the process of atrial septal puncture; 3. During the ablation procedure; 4. During the unconventional use of intravenous isoproterenol administration after ablation to identify potential trigger sites; 5. Paradoxical embolism. It is currently believed that anesthetic, sedative, and analgesic medications can cause vasodilation leading to a decrease in BP, resulting in transient abnormalities in the ST-segment of the ECG. In our hospital, general anesthesia is routinely used for atrial fibrillation radiofrequency ablation surgery. However, the ST-segment elevation in the inferior wall of this patient occurred approximately 30 minutes after anesthesia, so it is not considered to be related to anesthesia. Previous studies have confirmed that electrocardiographic abnormalities can occur during radiofrequency ablation for atrial fibrillation, with possible causes speculated to be: (1) catheter ablation in the left atrium stimulating autonomic ganglia, altering the balance of sympathetic and parasympathetic tone, which may lead to coronary artery spasm;^[2,3]^ (2) radiofrequency energy causes direct thermal injury or progressive inflammation to the coronary arteries, leading to vasospasm or blockage.^[4,5]^ However, ablation had not yet started in this patient. In 2014, Kumar et al reported a case of ST-segment elevation on the ECG caused by coronary artery spasm induced by isoproterenol during electrophysiological testing. The authors believed that it might be related to the sympathomimetic activity of isoproterenol, and dysfunction of the autonomic nervous system could lead to coronary artery spasm.^[6]^ This patient did not receive isoproterenol, so it is not considered as a factor. In 2015, Hayiroğlu reported a case of acute myocardial infarction with pulmonary embolism caused by patent foramen ovale, which was considered to be paradoxical embolism^.[7]^

Some literature reports that atrial septal puncture can cause transient ST-segment elevation. However, usually, patients have no obvious symptoms, and the incidence rate is approximately 1.3% to 5.2%, which does not affect the surgical process.^[8]^ The potential mechanisms for ST-segment elevation on the ECG during atrial septal puncture may include the following 4: 1. Vasospasm caused by imbalanced autonomic innervation; 2. Hypoperfusion of the right coronary artery resulting from reflex actions similar to the Bezold–Jarisch reflex; 3. Air embolism; 4. Allergic reaction to contrast agent.^[9–11]^ In this patient, examinations such as angiography clearly indicated that spasm occurred on the basis of coronary artery stenosis, leading to serious complications. It is currently believed that the atrial septum is rich in vagal efferent fibers, and long-sheath manipulation during or after atrial septal puncture may induce excessive vagal excitation. Vagal nerve excitation can lead to coronary artery spasm through multiple synergistic mechanisms: 1. Direct cholinergic activation: releases acetylcholine, which acts on M receptors (particularly M_3_ subtype) on vascular smooth muscle cells, triggering contraction; 2. Autonomic imbalance: excessive vagal stimulation inhibits sympathetic β_2_ receptor-mediated vasodilation, while potentiating α receptor-mediated vasoconstriction; 3. Enhanced calcium sensitivity via Rho-kinase pathway: vagal activation upregulates Rho-kinase signaling, increasing smooth muscle sensitivity to calcium and sustaining vasospasm.^[12,13]^ The reason for this phenomenon remains unclear. It may be because the right coronary artery is more richly innervated by vagal nerve fibers compared to the left coronary artery. The right coronary artery is more prone to cholinergic constriction and vasospasm, leading to ischemia in the inferior wall. ST-segment elevation is a rare complication of catheter ablation for atrial fibrillation during atrial septal puncture. The most likely mechanism for this phenomenon is coronary artery spasm caused by excessive vagal tone, which generally does not lead to serious complications and does not affect the surgery. However, if the patient has severe coronary artery stenosis, it may lead to difficulty in correcting the spasm, resulting in subsequent serious complications, as in this patient’s case. However, there is currently no effective method to prevent the occurrence of such coronary artery spasms. The lesson learned from this case is that atrial septal puncture can lead to coronary artery spasm, especially in the presence of coronary artery stenosis. This patient, however, is also unique. Throughout the entire disease course, the patient did not complain of chest pain or chest tightness, and had no risk factors for coronary heart disease such as hypertension, hyperlipidemia, and diabetes. But this also highlights the necessity of preoperative routine coronary CT examination. In summary, during atrial fibrillation surgery, continuous attention should be paid to changes in BP and ECG to promptly detect alterations and conduct differential diagnoses. For patients with confirmed coronary artery spasm, the administration of vasodilators such as nitroglycerin or nicorandil, and if necessary, the implantation of temporary pacemakers, intra-aortic balloon pump, extracorporeal membrane oxygenation, and other devices are effective treatment measures.

*Limitations*: This case is a case report, and the ECG changes caused by atrial septal puncture need to be analyzed specifically based on the patient’s condition during the operation.

## Author contributions

**Conceptualization:** Tao Zhou.

**Data curation:** Ping Gong, Yong Da Zhang, Yuan Zhi Zhou, Tao Zhou.

**Funding acquisition:** Tao Zhou.

**Investigation:** Ping Gong, Yong Da Zhang, Tao Zhou.

**Project administration:** Tao Zhou.

**Resources:** Tao Zhou.

**Supervision:** Ping Gong, Tao Zhou.

**Writing – original draft:** Tao Zhou.

**Writing – review & editing:** Tao Zhou.
